# Coxsackievirus B4 (CV-B4) linked to four severe neonatal infections including one fatal, Northern Ireland, October 2024 to February 2025

**DOI:** 10.2807/1560-7917.ES.2025.30.21.2500317

**Published:** 2025-05-29

**Authors:** Susan A Feeney, Marie Clare Crilly, Sharon Christie, Julie Lewis, Anne Eilis McColgan, Marc Niebel, James P McKenna

**Affiliations:** 1 Regional Virus Laboratory, Royal Victoria Hospital, Belfast, Northern Ireland, United Kingdom; 2 Paediatric Infectious Disease, Royal Belfast Hospital for Sick Children, Royal Victoria Hospital, Belfast, Northern Ireland, United Kingdom; 3 Paediatric Unit, Southern Health and Social Care Trust, Belfast, Northern Ireland, United Kingdom

**Keywords:** Enterovirus, Coxsackievirus B4, CV-B4, congenital, neonatal sepsis, coagulopathy, enterovirus meningitis, notifiable

## Abstract

We report four cases of severe neonatal non-polio enterovirus infection, requiring hospital admission. Infants presented to different regional hospitals across Northern Ireland from late 2024 to early 2025. Three had neonatal meningitis and one showed rapid demise, leading to sepsis, multi-organ failure and death at day 6 of life. All infants were less than 18 days old at presentation, two were considered congenital infections, with one presenting symptoms at birth. All infants had detectable enterovirus RNA, typed as coxsackievirus B4.

Severe and fatal non-polio enterovirus (NPEV) neonatal infections are rare, yet post-COVID-19, the United Kingdom (UK) and other European countries reported on cases and clusters of severe NPEV neonatal infection including myocarditis, sepsis and meningitis. The World Health Organization and the European Centre for Disease Prevention and Control issued reports in 2023 on observed increased case numbers of severe EV infection to direct public health attention to the risk; the viruses in circulation at this time included coxsackieviruses (CV-B3, CV-B4) and echovirus (E-11) [1-3]. Here, we describe four cases of neonatal NPEV infection presenting at an age range of birth to day 18, detected between October 2024 and February 2025, and managed at different hospital sites across Northern Ireland.

## Surveillance of non-polio enterovirus infections 

Northern Ireland has a total of five hospital Trusts. The Regional Virus Laboratory is in the Belfast Trust and is responsible for all enterovirus (EV) testing, including NPEV and poliovirus (Figure). The average number of EV real-time reverse transcription PCR (RT-qPCR) screens per year is approximately 8,000, with an 8–10% positivity rate for NPEV. Genotyping is performed at the reference laboratory of the United Kingdom Health Security Agency (UKHSA), London via partial VP1-gene sequencing. 

**Figure:**
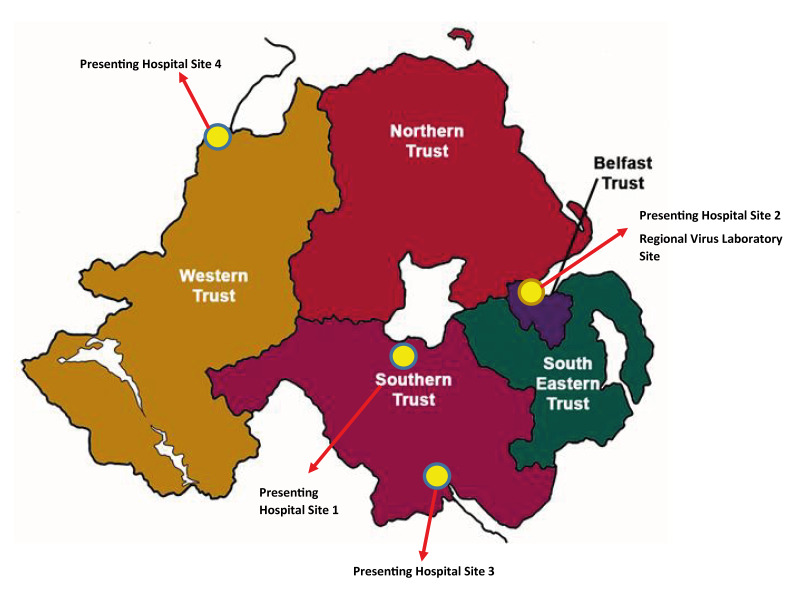
Location of Health Trusts and hospitals notifying cases of neonatal non-polio enterovirus (NPEV) infection, Northern Ireland, October 2024–February 2025 (n = 4)

Each yellow circle highlights the hospitals in which each of the four cases had presented when unwell. There was no epidemiological link established between these four cases.

The NPEV cases discussed here presented to four different hospitals in Northern Ireland (Figure). Non-acute flaccid paralysis/acute flaccid myelitis NPEV are not notifiable through the UK statutory notification system (Notifications of Infectious Diseases; NOIDS). Irrespectively, the Regional Virus Laboratory notified the local Public Health Agency of Northern Ireland in April 2025.

## Case presentation

All infants (Infants A–D, presented in order of severity) were born close to term, all via elective Caesarean section, in good condition with Apgar scores of 9 and 9 at 1 min and 5 min, respectively, and good weight. One infant (Infant A) quickly deteriorated postnatally with severe outcome and death. Infants B–D, all discharged after birth, were each re-admitted between day 3 and 18. 

Infants A and B showed signs of infection in the first 7 days of life, which is regarded as congenital infection [4,5]; these were the two most severe cases of the four. Infants C and D had a later onset and a milder presentation, with both discharged with a primary diagnosis of enterovirus meningitis. There was no epidemiological link between any of the four cases mentioned. The characteristics of the four cases are presented in Table 1.

**Table 1:** Characteristics of infants presenting with neonatal enterovirus infection, Northern Ireland, October 2024–February 2025 (n = 4)

Characteristics	Infant			
	A	B	C	D
Date of birth	Late Dec 2024	Late Jan 2025	Late Sep 2024	Late Dec 2024
Sex	Female	Male	Male	Male
Gestation (weeks + days)	39 + 3	39 + 0	39 + 3	39 + 3
Birth type	Elective caesarean section	Elective caesarean section	Elective caesarean section	Elective caesarean section
Birth weight (g)	3,773	3,630	4,415	3,265
Age at symptom onset	Birth	3 days	17 days	12 days
Age at hospital admission	Birth	4 days	18 days	13 days
Hospital location^a^	Site 1	Site 2, Site 3	Site 2	Site 4
Symptoms	Skin rash, seizures, deranged LFT, coagulopathy	Apnoea, lethargy, respiratory distress, coagulopathy, thrombocytopenia	IWOB, fever (>38 C^◦^), unsettled, mottled appearance	Fever (38–39 C^◦^), reduced feeding, lethargy, increase in soiled nappies
Antimicrobials	IV aciclovir, IV benzylpenicillin, IV gentamicin	IP amoxicillin	IV amoxicillin, IV cefotaxime	IV amoxicillin, IV cefotaxime
**Laboratory investigation**				
Peak LFT AST/ALT U/L (normal range: 0–40 U/L)	178/152	388/82	24/20	Not tested
Peak CRP mg/L (normal range: 0.0–5.0 mg/L)	8.92	10.8	43.8	16.3
Lowest platelet count (normal range: 160–500 x10^9/L)	32	8	303	322
Coagulation screen (prothrombin time) 10–14.2 s	25.5	15	12.6	Not tested
Respiratory PCR (panel^b^; NPEV^c^)	Panel neg.; NPEV-pos.^d^	Panel neg.; NPEV-pos.^d^	All neg.	Not tested
Viral meningitis PCR (panel^e^; NPEV^c^)	Panel neg.; NPEV-pos.^f,g^	Panel neg.; NPEV-pos.^f,h^	Panel neg.; NPEV-pos.^f^	Panel neg.; NPEV-pos.^f^
Bacterial meningitis PCR (panel^i^; NPEV^c^)	All neg.	All neg.	All neg.	All neg.
Cytomegalovirus PCR	Neg.	Not tested	Neg.	Not tested
Gastrointestinal (GI) PCR (panel^j^; NPEV^c^)	Panel neg.; NPEV-pos.^g^	Not tested	Not tested	Not tested
Toxoplasma serology	Neg.	Neg.	Not tested	Not tested
Syphilis serology	Neg.	Neg.	Not tested	Not tested
Parvovirus B19 serology	Not tested	Neg.	Not tested	Not tested
**Clinical outcomes**				
Potential risk factors for infection	Mother unwell, ILI 2 days before delivery; sibling unwell, ILI in week before delivery	Sibling with recent respiratory illness	Sibling with recent gastroenteritis	Sibling at home, no reports of recent illness
Age at discharge	Not discharged; died	13 days	23 days	16 days
Age at death	6 days	NA	NA	NA
Final diagnosis	Enterovirus meningitis, multiorgan failure	Enterovirus meningitis	Enterovirus meningitis	Enterovirus meningitis

ILI: influenza-like illness; IP: intraperitoneal; IV: intravenous; IWOB: increased work of breathing; LFT: liver function tests; neg.: negative; NPEV: non-polio enterovirus; pos.: positive.

^a^ Site 1 is located in the Southern Trust, Site 2 in the Belfast Trust, Site 3 in the Southern Trust and Site 4 In the Western Trust (Figure).

^b^ Respiratory panel: COVID-19, influenza A/B, respiratory syncytial virus (RSV), human rhinovirus (HRV), coronavirus (CoV), parainfluenzavirus (PF), adenovirus (ADV), metapneumovirus, C pneumonia and mycoplasma pneumonia (all in-house/laboratory-developed tests) (all PCR-negative).

^c^ Enterovirus was detected by an in-house/laboratory-developed assay (pan-enterovirus TaqMan-based RT-qPCR).

^d^ Respiratory secretions.

^e^ Viral meningitis panel: herpes simplex virus type 1 and 2, varicella zoster virus (Altona), and an in-house parechovirus (PeV) (all PCR-negative).

^f^ Cerebrospinal fluid sample.

^g^ Stool/faecal sample.

^h^ Blood sample.

^i^ Bacterial meningitis panel: *Neisseria meningitidis, Streptococcus pneumoniae, Haemophilus influenza* (Bosphore), and an in-house Group B Streptococcus (all PCR-negative).

^j^ Gastrointestinal (GI) panel: norovirus, rotavirus, astrovirus, adenovirus, sapovirus (EntericBio) (all PCR-negative).

## Molecular investigation

Enterovirus was detected in samples from all four infants using an in-house laboratory-developed 5’UTR RT-qPCR assay. Enterovirus-positive sample types included respiratory secretions, blood, cerebrospinal fluid (CSF), faeces and skin surface swabs (Table 2). All the EV-positive samples of sufficient volume were referred to UKHSA in London, United Kingdom (UK) for EV-VP1 typing by Sanger sequencing and were subtyped as species B, coxsackievirus B4 (CV-B4) [6]. Unfortunately, next generation sequencing (NGS) was not available from the reference laboratory on these clinical samples; therefore it has not been possible to generate phylogenetic analysis. 

As part of the viral and bacterial meningitis panel testing on site, all available samples (swabs and/or CSF) were also processed for herpes simplex virus type 1 and 2, varicella zoster virus, pathogens causing bacterial meningitis and Group B Streptococcus by PCR and were negative; available faeces samples were tested using the gastrointestinaI PCR panel and were negative for all pathogens (with the exception of EV) (Table 1). Available respiratory samples were tested for the full respiratory screen and were negative for all pathogens (with the exception of EV). Blood cultures on all four infants were negative.

All four infants had EV-positive samples confirmed as genotype CV-B4 by partial sequencing of the VP1-gene. Samples from these four infants were the only CV-B4 genotypes in the year period from March 2024 to March 2025, and presentation occurred relatively close in time (Oct 2024–Feb 2025).

**Table 2:** Results of molecular investigation of infants testing positive for enterovirus coxsackievirus B4, Northern Ireland, October 2024–February 2025 (n = 4)

Variable	Infant							
	A^a^				B^b^		C	D
Sample type	Secretions 1	Stool	Secretions 2	CSF	CSF	EDTA blood	CSF	CSF
Ct value	26.73	28.84	34.59	34.8	30.56	24.90	33.78	32.64
Typing result	CV-B4 (partial VP1)				CV-B4 (partial VP1)		CV-B4 (partial VP1)	CV-B4 (partial VP1)
Outcome	Admitted, NICU, EV sepsis, deceased				Admitted, NICU, EV meningitis, discharged		Admitted, PW, EV meningitis, discharged	Admitted, PW, EV meningitis, discharged

CSF: cerebrospinal fluid; Ct: cycle threshold; CV-B4: coxsackievirus B4; EV: enterovirus; NICU: neonatal intensive care; PW: paediatric ward.

^a^ Secretions were collected at two timepoints 3 days apart. Samples from Infant A (groin and umbilical skin surface swabs) were EV-positive but not typed as Ct values were too high.

^b^ Infant B respiratory secretions were EV-positive but not typed as Ct values were too high.

## Case reports

### Infant A

At the point of delivery, Infant A had blisters on the dorsum of both hands and was admitted to the hospital Site 1’s neonatal unit. The infant was treated with aciclovir and first-line antibiotics (benzylpenicillin and gentamicin) as per NICE clinical guidelines CG149 [7]. The infant was stable and breastfeeding but by day 3 had a widespread erythematous rash and had undergone a generalised tonic-clonic seizure. The infant received a loading dose of phenobarbitone and had become tachycardic and tachypnoeic. The infant had a second seizure episode overnight, and with prolonged apnoea and bradycardia. Liver function became deranged (AST: 178 U/L; ALT: 152 U/L). The infant developed coagulopathy and thrombocytopenia and required fresh-frozen plasma and platelet transfusions. On day 4, Infant A was commenced on inotropes because of falling blood pressure, initially dopamine and then adrenalin infusions but became increasingly hypotensive. Echocardiography was performed and this showed a structurally normal heart, mildly reduced function and marked biventricular hypertrophy. By day 5, EV RNA was confirmed as coxsackievirus B4 (CV-B4) by RT-qPCR from an umbilical swab, respiratory secretions, skin surface swabs, cerebrospinal fluid (CSF) and faeces (Table 2). In the early hours of day 6, the infant developed metabolic acidosis with increasingly poor perfusion, showed no movements including no breathing movements and there was evidence of multiorgan failure. Infant A passed away on day 6 of life.

### Infant B

Infant B presented to a different district general hospital (Site 2) at 4 days of age with profound apnoea, lethargy and fatigue, and a mottled appearance. The infant was transferred on day 4 after admission to the neonatal intensive care unit at Site 3. Enterovirus RNA was confirmed by RT-qPCR as CV-B4 from CSF and EDTA blood (Table 2). The infant required high-flow oxygen and had thrombocytopenia, requiring a total of five platelet transfusions. The infant was commenced on intraperitoneal amoxicillin. Liver function became deranged (AST: 388 U/L; ALT: 82 U/L; CRP: 10.8 mg/L). An ultrasound of the abdomen was performed with the liver demonstrating heterogeneous echo pattern and no definite hepatomegaly. Cranial ultrasound and echocardiogram observed normal function. Infant B began to improve, allowing discharge home by day 13.

### Infants C and D

Infants C and D were born at different hospital sites (Site 2 and 4), returning to the hospital at 18 days and 13 days of age, respectively. Infants presented as pyrexic and unsettled; Infant C showed increased work of breathing and a mottled appearance; Infant D was lethargic with reduced feeding. Both had elevated C-reactive protein and white cell count, both were commenced on intravenous cefotaxime and amoxicillin. Cerebrospinal fluid analysis showed reduced glucose, elevated protein levels, and RT-qPCR-detectable EV RNA (Table 2). Infant C’s urine culture showed detectable *Klebsiella oxytoca* and Infant D’s urine culture showed detectable *Enterococcus* sp. Both were discharged by 5 days after admission with a primary diagnosis of EV meningitis and both infants were confirmed positive for CV-B4. 

## Discussion

Enterovirus infections in neonates are common and can present as asymptomatic or as any combination of fever, irritability, lethargy, poor feeding or rash. However, when contracted in the first few days of life, symptoms can be very severe and are known to cause sepsis, meningoencephalitis, myocarditis, coagulopathy, hepatitis and meningitis, often leading to fatality [4,5]. 

Enterovirus can be transmitted via respiratory and faecal-oral routes, but can also be acquired vertically, peripartum or horizontally in the neonate [8-10]. Thrombocytopenia, derangement of liver function or coagulopathy should cause suspicion of EV in neonates. Severe infection is likely the result of an infant’s immature and naive immune system, and insufficient maternal protective immunoglobulin transfer. The infected neonate is at greatest risk for severe morbidity and mortality when signs and symptoms develop in the first few days of life. 

An additional risk factor for severe neonatal infection is the EV serotype. CV-B4 belongs to the EV genus species group B, family Picornaviridae. CV-B4 is recognised as a known cause of meningitis and other neurological disorders with a commonly proposed route being the entry to the CNS via infected mononuclear cell penetration of the blood–brain barrier and invasion of the choroid plexus epithelium [9,10].

At present, there is no specific antiviral for EV; treatment is supportive and primarily focussed on the management of complications. With severe cases, intravenous immunoglobulin (IVIG), intravenous steroids, investigational antivirals (e.g. Pocapavir V-073) and immunomodulation (e.g. anakinra) have been used [11]. Intravenous immunoglobulin, although titres are not standardised, may potentially improve mortality if started early in infection [11]. The ‘compassionate use only’ antiviral, Pocapavir, is a capsid inhibitor specific to Picornaviridae, and blocks viral uncoating and thus release of viral RNA into the host cell. There are a small number of case reports on Pocapavir use, including neonatal sepsis [12,13]. It would seem a combined approach of IVIG and an antiviral may be the best approach to adopt in cases of severe and life-threatening EV infection. Early initiation of treatment is key, but depends on early clinical suspicion and investigation of EV as the causal agent. Together with issues in accessing antivirals and no clear framework on use, treatment with these ‘compassionate use’ medications is difficult.

With the exception of acute flaccid paralysis or acute flaccid myelitis, NPEV infection is not notifiable in many countries, and severe infection may go unreported [14]. The need for widespread robust clinical diagnostics and epidemiological surveillance is apparent, both from a European and a global perspective [15-17]. With the advent of whole genome sequencing and next generation sequencing, the ability to discriminate beyond partial VP1 sequencing for EV can only be an advantage in global monitoring. These RNA viruses have the potential for sequence modification and mutation, resulting in new viruses and new lineages that may potentially have pathological impact. Centralised data collection systems for monitoring circulating EV sequences, such as NextStrain, would potentially have many advantages such as data sharing, tracking of global epidemics, identification of new or re-emerging strains, informing diagnostics, informing public health initiatives and benefiting therapeutic initiatives. 

## Conclusion

Early suspicion of EV infection as a differential diagnosis, with timely and supportive laboratory diagnosis, may enhance therapeutic intervention. Enhanced surveillance data at the molecular level can inform on circulation of changing lineages and/or new subtypes and hence builds knowledge on associated pathogenesis.

## Data Availability

No phylogenetic data were generated.
